# Effects of Flat-Side Design on Torsional and Bending Stress of Nickel–Titanium File by Finite Element Analysis

**DOI:** 10.3390/bioengineering13060600

**Published:** 2026-05-22

**Authors:** Yinjie Yang, Xinfang Cao, Jiwu Zhang, Yuqing Zhou, Songhao Chen, Benxiang Hou

**Affiliations:** 1 Center for Microscope Enhanced Dentistry, Beijing Stomatological Hospital, Capital Medical University, Beijing 100162, China; 2 School of Biomedical Engineering, Shanghai Jiao Tong University, Shanghai 200030, China; 3 Department of Mechanics, School of Mechanics and Engineering Science, Peking University, Beijing 100871, China

**Keywords:** endodontics, finite element analysis, nickel–titanium, root canal preparation, stress, mechanical

## Abstract

Background: This study evaluated the effects of flattening the side of the bending resistance and torsional resistance of nickel–titanium files through finite element analysis of a novel flattened file and a standard nonflattened file. Methods: For torsion analysis, the tip of the file was fixed at 3 mm, generating a torque of 2.5 N·mm at the handle. For bending analysis of curved root canals (45° and 60°), the handle was kept fixed, a force of 1 N was applied at the tip, and the file was fixed at 3 mm. Results: The standard nonflattened file exhibited better torsional resistance. In contrast, the novel flattened file showed improved flexibility under 45° bending. Under this condition, lower maximum von Mises stress was observed in the flattened design compared with the standard file. At 60° bending, stress distribution varied with loading orientation, and higher stress concentrations were observed in the flattened file under specific bending directions, indicating reduced bending resistance under large deformation conditions. Conclusions: Since lateral flattening may reduce the cyclic resistance of files, caution should be exercised in the clinical use of such files.

## 1. Introduction

The main goal of root canal therapy is to remove infected or necrotic pulp tissue and bacteria, while maintaining a well-sealed root canal system to prevent reinfection [1,2]. Therefore, shaping and cleaning the root canal are key steps toward achieving the intended goal. However, achieving these objectives is challenged by several clinical limitations associated with endodontic instruments. Owing to the inherent rigidity, stainless-steel files are prone to various complications, including procedural errors such as perforation and canal deviation [3]. Nickel–titanium (NiTi) rotary instruments are widely used in root canal therapy. Compared to stainless-steel files, NiTi files have shorter shaping times and better cutting performance [4]. In addition, the flexibility of NiTi files helps reduce the incidence and severity of complications during root canal therapy. However, in clinical practice, the use of motor-driven NiTi files is often complicated by instrument separation, particularly during canal shaping in narrow and severely curved root canals [5,6].

Fractures of NiTi files are caused by cyclic fatigue and torsional overload [7,8]. Cyclic fatigue fractures occur due to the repeated generation of compressive and tensile stresses in curved root canals when the file is rotated. In clinical settings, this type of fracture is particularly challenging owing to its unpredictable occurrence. Conversely, torsional fractures take place when the file continues to rotate while its tip is jammed in a narrow root canal. File fracture during root canal shaping presents clinicians with a complex clinical challenge. Fragment retrieval is often laborious and time-intensive, and may cause extensive dentine removal, thereby elevating the risk of tooth fracture and worsening the overall prognosis [9].

To prevent file fracture, multiple approaches have been explored, including thermal processing of NiTi alloys, improvements in motion techniques, and alterations in instrument design [10,11]. Cross-sectional design plays a key role in defining the stiffness and flexibility of NiTi files, which are directly related to torsional resistance. Thus, manufacturers have adjusted this shape to achieve better flexibility and higher resistance to fracture. Cross-sectional shapes commonly include triangular, square, rectangular, and S-shaped [12]. Studies have shown that the torsional fracture resistance of U-shaped files and triangular cross-sectional files is lower than that of square, convex triangular, and S-shaped files. The reduced core diameter, together with the deep groove configuration, is considered responsible for this behavior. Moreover, the central core of NiTi files affects bending stiffness, which subsequently influences cyclic fatigue resistance [13].

In recent years, several dental companies have launched novel heat-treated rotary nickel–titanium instruments with a flat-edge design [14]. This innovative geometry has been the subject of comprehensive investigation across multiple studies, examining both its mechanical performance characteristics and clinical implications. Research has demonstrated that blade geometry—whether nonflattened, flat, or hybrid—substantially influences the mechanical properties of NiTi instruments, with metallurgical composition playing a critical complementary role in determining instrument behavior under clinical conditions [15]. Dynamic photo elastic analyses have further elucidated the stress distribution patterns associated with heat-treated flat-side rotary instruments, providing valuable insights into how these designs interact with canal anatomy during instrumentation [16]. Additionally, preliminary ex vivo investigations have explored whether the flat-side design in engine-driven full-sequence file systems offers advantages in terms of root canal disinfection efficiency [17]. Collectively, these studies suggest that flat-edge instruments, particularly when combined with advanced heat treatment technologies, may offer improved mechanical performance and stress management compared to traditional designs, though ongoing research continues to optimize their clinical applications. All manufacturers generally claim that these instruments’ flat-edge designs offer numerous advantages over traditional, non-flat instruments. Owing to the flat cross-section, the instrument contains less metal mass, theoretically resulting in enhanced flexibility and cyclic fatigue resistance. Additionally, the improved design is reported to prolong fatigue life and lessen blade engagement, while the longitudinal flat surfaces could facilitate debris evacuation from the grooves to the relief area, thereby potentially improving cleaning performance. Contrary to these claims, a recent comprehensive analysis comparing the performance of flat-edge instruments with traditional non-flat-edge instruments using a multi-method evaluation revealed inferior performance in various areas, including cyclic fatigue, maximum torque, rotation angle, maximum flexion strength, and flexibility [15,16]. The study also highlighted manufacturing inconsistencies in some flat-edge instruments, such as segment discontinuities, varying orientations, and issues related to heat treatment. In addition, previous investigations have reported inconsistent results concerning the performance of flat-edge instruments. For example, Ubaed and Bakr [17] found that the AF F-One (Fanta Dental Material Co., China) exhibited lower resistance to cyclic fatigue after clinical use, compared to the 2Shape (Micro Mega, France) instrument, while Mogahed et al. [18] observed that the AF F-One produced more debris compared to the One Shape (Micro Mega, France) and One Curve (Micro Mega, France) systems but demonstrated superior cleaning ability. Conversely, Di Nardo et al. [19] reported that flat-faced instruments exhibited better forming capabilities and improved resistance to bending fatigue compared to non-flat prototype instruments.

Further exploration of the flat-sided concept of endodontic instruments remains important. Existing experimental studies have evaluated torsional and cyclic fatigue performance of flat-side instruments, but the stress distribution produced by side flattening under controlled torsional and direction-specific bending loads has not been fully clarified. Therefore, this study aimed to compare the maximum von Mises stress distribution of side-flattened and nonflattened NiTi files with similar design features under simulated torsional and bending loading conditions. The null hypothesis was that side flattening would not alter the maximum von Mises stress distribution of NiTi files under the simulated conditions.

## 2. Materials and Methods

### 2.1. Designing Files and Root Canals

**Figure 1:**
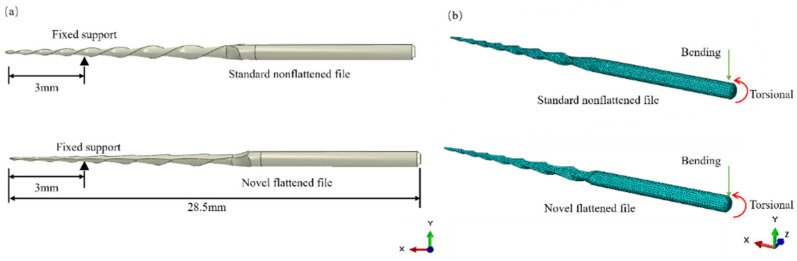
Models: (**a**) CAD model (standard nonflattened file and novel flattened file); the flattened side is visible in the cross-sectional view of the novel file. (**b**) Finite element mesh model and boundary conditions.

Two different of ideal NiTi rotary instruments were designed by computer-aided engineering software (SolidWorks, 2016, Massachusetts, USA): a standard nonflattened file and a novel flattened file (Figure 1). The design is based on two NiTi instruments produced by the same manufacturer (Bondent, Shanghai, China). The taper of all instruments is 6%, and the tip size is 0.25 mm. The cross-sectional areas of both files are S-shaped, but only the novel flattened file has slightly flattened sides.

### 2.2. Finite Element Model

The mechanical behavior of NiTi files was numerically analyzed using Abaqus (Version 6.14) software to simulate their bending and torsional resistance. These values, along with the cross-section and taper of the tool, determine the material’s strength. The material properties used in the simulation were taken from the study of Díaz-Flores García V. et al. [20] and loaded into the software, as shown in Table 1. The Auricchio material model was used to simulate the hyperplastic behavior of the NiTi alloy, where EA and EM represent the Young’s modulus of austenite and martensite, respectively. Ten-node tetrahedral elements were chosen to discretize the model. They are particularly well-suited for modeling irregular meshes. The final node and element numbers of the two finite element models were determined (Table 2). For the non-flat file, the mesh contained 11,008 nodes and 5521 elements. For the flat file, 12,104 nodes and 5688 elements were required. The boundary conditions used to simulate the behavior of root canal treatment instruments comply with ISO 3630-1 [21].

The analyses of the two groups are as follows: (1) Torsion analysis and von Mises stress distribution at 3 mm from the tip. A support point was placed at this millimeter and a torque of 2.5 N·mm was applied to the handle to rotate the file around its longitudinal axis. (2) Bending analysis in curved tubes (45° and 60°). The nickel–titanium instrument was fixed at a distance of 3 mm from the tip, effectively limiting its displacement in three axes (X, Y, and Z axes). Displacement was applied to the instrument handle in a specific direction to obtain inclination angles of 45° and 60°. Since the bending resistance of the novel flat root canal file cannot be fully evaluated along a single direction along its cross-section, three directions were selected for comprehensive evaluation of the bending resistance of the novel flat root canal file: parallel to the flat surface, in the same direction as the normal to the flat surface, and in the opposite direction to the normal to the flat surface.

## 3. Results

### 3.1. Torsion Test

**Figure 2:**
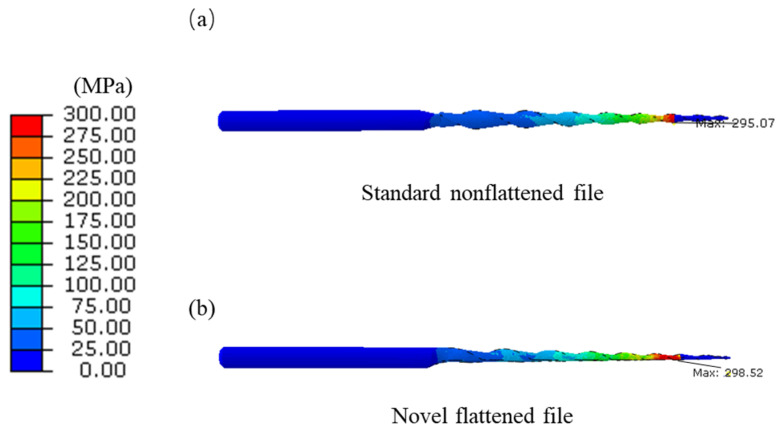
Torsion test results: (**a**) von Mises stress distribution of the standard nonflattened file to torsion test at 2.5 N·mm; (**b**) von Mises stress distribution of the novel flattened file to torsion test at 2.5 N·mm. The color scale indicates the magnitude of von Mises stress in MPa.

**Figure 3:**
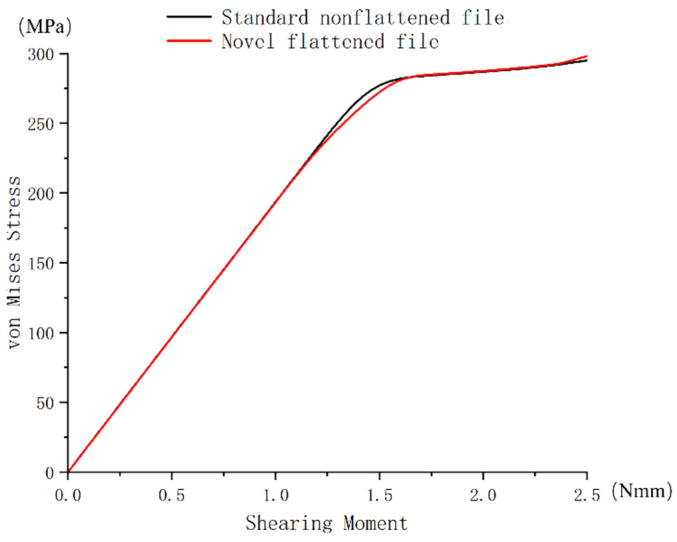
Torsion test results: graph representing the stress distribution to torsional moment.

In the torsion comparison of the two files, we observed that the flat file experienced the least von Mises stress in the analysis (Figure 2). For each file, the highest stress was found near the fixed portion. The maximum stress of the standard non-flat file is 295.07 MPa. By comparison, the flat-sided file shows a slightly higher value of 298.52 MPa (Table 3). As shown in Figure 3, the stress trends generated during the torsion process as the torque changes are similar. Therefore, the flat-side design does not significantly reduce the torsional resistance performance of the nickel–titanium file.

### 3.2. Bending Test

**Figure 4:**
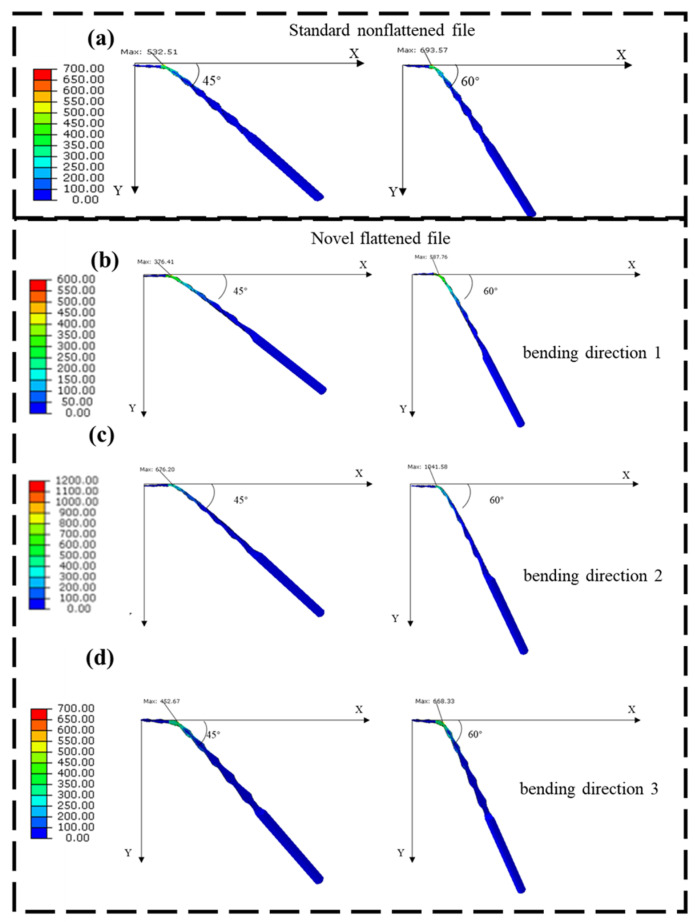
Results of the bending test: (**a**) von Mises stress distribution at 3 mm from the tip of the standard file; (**b**) von Mises maximum stress distribution at 3 mm from the tip when the flat file is bent along direction 1; (**c**) von Mises maximum stress distribution at 3 mm from the tip when the flat file is bent along direction 2; (**d**) von Mises maximum stress distribution at 3 mm from the tip when the flat file is bent along direction 3.

**Figure 5:**
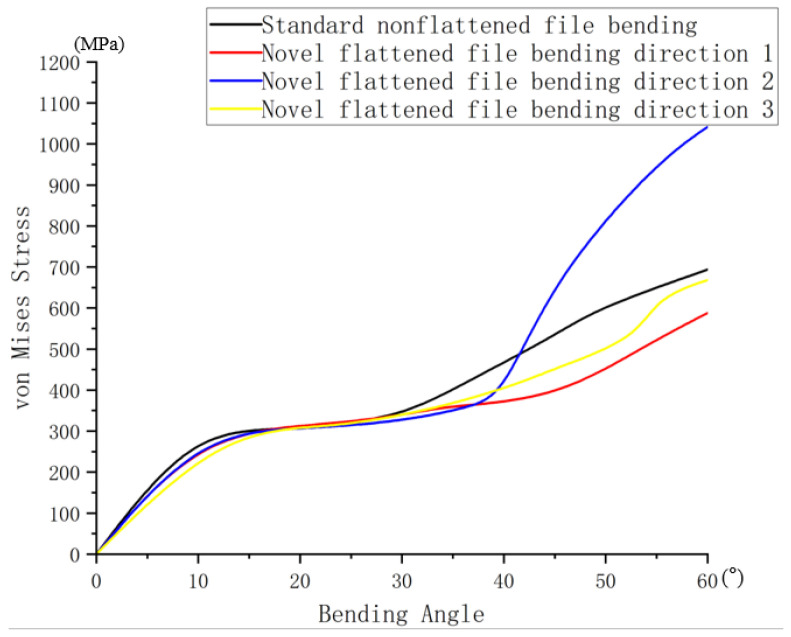
Bending test results: graph representing the stress distribution with respect to the bending angle.

Both of these files are bent at a 45-degree angle and a 60-degree angle in accordance with the provisions of the ISO 3630-1 standard. Stress distribution assessment shows that, under a fixed bending angle of 45°, the maximum von Mises stress of the non-flat file is 532.51 MPa. The maximum stresses generated by applying displacement along three directions, namely, in the same direction as the normal to the flat surface (bending direction 1), in the opposite direction to the normal to the flat surface (bending direction 2), and parallel to the flat surface (bending direction 3), until the flattened file was bent to 45 degrees, were 376.41 MPa, 676.20 MPa, and 452.67 MPa, respectively. When bent to 60 degrees, the stress along direction 2 increases rapidly to 1041.58 MPa. The stresses in the other two directions are 587.76 MPa and 668.33 MPa, respectively. For a standard file, the maximum stress value is 693.57 MPa, which is slightly greater than the stress along directions 1 and 3 of a flat file, but much less than the stress along direction 2 (Figure 4; Table 3). The conclusion that can be drawn is that the novel flattened file can improve its bending resistance when bent at a specific angle, regardless of the direction along the novel flattened file. However, when bent to approximately 60 degrees, the maximum stress caused by bending in the opposite direction of the normal to the flat surface increases rapidly, indicating a more pronounced stress localization under this loading condition (Figure 5).

## 4. Discussion

Finite element analysis (FEA) can directly evaluate the impact of different file structures on their mechanical properties through a series of computational operations. This makes it possible to calculate stress distribution and concentration, minimize uncontrollable variables, and repeat analyses under the same test conditions. Using FEA to study the performance of rotary files in root canals can save time and resources in the development of new instruments. The images generated by the computer software used in this study have potentially important clinical applications. They can visually show the areas of the file most prone to complications, thus helping to improve the safety of dental treatments [22,23].

Recently, a new type of flat-sided nickel–titanium file has emerged. Manufacturers claim that this design reduces the contact area with the root canal, enhances debris removal, and improves flexural strength. Since understanding how structural changes (such as flat sides) affect the physical properties of nickel–titanium files is helpful for their further development, this study compares the physical properties of nickel–titanium files with and without flat sides, using the same cross-sectional area. The novel flattened file instruments and their corresponding standard nonflattened file instruments used in this study were both manufactured by the same company, confirming that the manufacturing processes of the two instruments are almost identical, differing only in the processing method of the flat sides. A 6% taper was selected in this study because it is a commonly used and clinically relevant intermediate taper in NiTi rotary systems, and it provides a balanced stiffness level for evaluating the effect of geometric differences on stress distribution under controlled conditions. Torsional strength is a crucial factor influencing the clinical performance of nickel–titanium files, especially in shaping narrow root canals. Previous studies have shown that torsional strength is affected by factors such as file size, cross-sectional shape, and alloy properties. One study found that a larger core area in the file cross-section corresponds to higher torsional strength. Increasing the number of threads on the file can improve stress distribution on the threads, thereby increasing torsional fatigue strength. In this study, no significant differences were observed in these values between the standard nonflattened file and the novel flattened file. Therefore, lateral flattening of nickel–titanium files does not affect their torsional strength.

Flattened side design makes the results of the bending test more direction-dependent. In fact, the side blade geometry of the flattened file disrupts uniform mechanical stress distribution, increasing the risk of deformation or breakage [16,24]. Under 45-degree bending, the flattened file showed lower maximum von Mises stress than the nonflattened file in directions 1 and 3, but higher stress in direction 2. Under 60-degree bending, direction 2 produced the highest maximum von Mises stress among all simulated bending conditions. These findings suggest that the flat-side geometry may reduce stress concentration under some controlled bending orientations while increasing stress concentration under others. Compared with the other tested instruments, the nonflattened file shows better performance in key mechanical parameters such as fracture time, maximum torque, and buckling strength. These results emphasize the capability of nonflattened instruments to endure high-stress environments, particularly in demanding clinical situations. The excellent performance of the nonflattened file highlights the mechanical advantages of its nonflattened blade design, which seems to allow for a more balanced distribution of mechanical stress on the instrument, thereby enhancing its structural integrity [25,26]. However, unlike the above results, at small bending angles, the flat design, due to its inherent geometry, exhibits slightly less bending in certain directions than the non-flat root canal [27]. However, at large bending angles, the flat-sided design shows markedly increased stress under the same deformation conditions, suggesting reduced mechanical tolerance in highly curved configurations. It is notable that the worst-case bending direction may not occur consistently in vivo because file orientation changes during rotation and depends on canal anatomy. However, the three directions were selected to evaluate directional anisotropy caused by the flat-side geometry, rather than to reproduce all possible clinical file orientations. However, it should be noted that the present finite element analysis does not directly assess fracture resistance, cyclic fatigue behavior, or clinical performance. The selected bending directions were designed as controlled worst-case simulations to evaluate geometric anisotropy rather than to replicate all possible in vivo file orientations. Future studies should therefore include experimental validation and investigate instruments with different tapers and clinical conditions to further verify the present findings.

Current evidence suggests that extreme caution is necessary when applying newly developed flat-side instruments in curved root canals because they may generate greater stress on root canal walls, leading to excessive deformation and possible reductions in performance and lifespan. Consequently, careful assessment, further modification, and additional validation studies are needed before routine clinical use. The study by Jeong et al. [14] further supports this suggestion, noting that the lateral flattening of nickel–titanium instruments for root canal treatment may reduce their cyclic fatigue resistance. The authors emphasize that caution must be exercised when using such instruments in clinical practice to reduce potential complications.

## 5. Conclusions

Under controlled conditions and within the limitations of this finite element analysis study, finite element analysis of flat and non-flat files yielded the following conclusions:

1. Flattening does not substantially reduce the torsional resistance of nickel–titanium files.
2. Compared to non-flat files, flat files exhibit greater flexibility when bent at 45 degrees, except in direction 2.
3. When the bend reaches 60 degrees, the stress resulting from bending in the opposite direction to the vertical plane is substantially greater than that of non-flat files and other bending directions of flat files, thus greatly reducing their bending resistance. The bending resistance of flat files decreases considerably at large bend angles.

## Figures and Tables

**Table 1:** Parameters used to describe the consecutive models for simulation.

Parameters	Value
Austenite Elasticity	55,737 MPa
Austenite Poisson’s Ratio	0.33
Martensite Elasticity	19,106 MPa
Martensite Poisson’s Ratio	0.33
Transformation Strain	8.6%
(*δσ*/*δT*) Loading	6.7
Start of Transformation Loading	448 MPa
End of Transformation Loading	511 MPa
Reference Temperature	22 °C
(*δσ*/*δT*) Unloading	6.7
Start of Transformation Unloading	161 MPa
End of Transformation Unloading	118 MPa

**Table 2:** Number of nodes and elements in the finite element models.

Model	No. of Nodes	No. of Elements
Standard nonflattened file	36,486	23,019
Novel flattened file	20,996	10,192

**Table 3:** Von Mises stress values at the nodes of a finite element model **(MPa)**.

Model	Standard Nonflattened File	Novel Flattened File	
Bending 45°	532.51	bending direction 1	376.41
		bending direction 2	676.20
		bending direction 3	452.67
Bending 60°	693.57	bending direction 1	587.76
		bending direction 2	1041.58
		bending direction 3	668.33
Torsional	295.07	298.52	

## Data Availability

No new data were created or analyzed in this study.
